# A Preliminary Study of Correlates of Premature Birth and Their Influence on Cortisol Levels in Young Children

**DOI:** 10.1177/10998004231209429

**Published:** 2023-10-20

**Authors:** Sophia Kloosterboer, Fabiënne Bertina Anolda Naber, Hiltje Heyman, Angelique Hoffmann-Haringsma, Tibor Markus Brunt

**Affiliations:** 1 Department of Pediatrics, 6993 Erasmus University Medical Center, Rotterdam, Netherlands; 2 Department of Psychiatry, 8124 University Medical Center Utrecht, Utrecht, Netherlands; 3 Het Kleine Heldenhuis, Rotterdam, Netherlands; 4 Department of Neonatology, 6992 St Fransiscus Hospital, Rotterdam, Netherlands; 5 Department of Psychiatry, 26066 Amsterdam University Medical Center, Amsterdam, Netherlands

**Keywords:** preterm children, cortisol, delivery route, prematurity, hypothalamic-pituitary-adrenal-axis

## Abstract

**Objective:**

The HPA-axis is programmed during early infancy, but a lot is unknown about the programming of the HPA-axis in prematurely born or small for gestational age (SGA) children. Therefore, the aim of this preliminary study was to investigate the influence of prematurity and variables associated with birth on cortisol levels in young children.

**Methods:**

Cortisol was measured in a cross-sectional design in 38 premature born participants (<37 weeks of gestation), aged between 3 – 9 years old. Correlates of prematurity (degree of prematurity and birth delivery route) were investigated in relationship with cortisol levels with regression analysis.

**Results:**

Corrected for sex, delivery by C-section was associated with lower cortisol levels in the children (*ß* = −.42, *p =* .028), with an explained variance of 34%.

**Conclusion:**

Birth delivery route by C-section is associated with lowered (or flattened) cortisol levels in children born prematurely. This is clinically relevant and might have important implications, because an HPA-axis disturbance might lead to developmental problems later on in life. However, future research is necessary to investigate the underlying indications for performing a C-section, which will help to understand factors that influence the HPA-axis development in children born prematurely.

## Introduction

Children born very prematurely (gestational age <32 weeks) have a high risk of developmental medical complications (Frey & Klebanoff, 2016). In the Netherlands, children that are born very prematurely are routinely followed-up by a pediatrician at the age of 6 months, 12 months, 24 months, 5 years, and 8 years (van der Pal et al., 2014). The outcomes of prematurity are multifactorial, determined by genetic factors, intra-uterine and peri-partum circumstances, neonatal care provided, and the home environment (Behrman & Butler, 2006). The neonatal phase, with environmental stimuli, is a crucial period for brain development and it has been shown that preterm infants are more susceptible to brain deficits in a review by Cheong et al. (2020). For instance, a large systematic review showed smaller brain volume and reduced grey and white matter in preterm infants (Keunen et al., 2012). Other data has shown that the adrenal cortex and specific areas of the brain surrounding the hypothalamus and pituitary gland (units of multiple hormonal-axes), and thereby the Hypothalamic-Pituitary-Adrenal axis (HPA-axis), were underdeveloped in preterm infants (Bolt et al., 2002). As the HPA-axis regulates corticosteroids, it has likewise been found that serum levels of cortisol were decreased in preterm and very low birth weight (VLBW) infants (<32 weeks gestational age/<1500 g birth weight; Hwang et al., 2019; Ng, 2016; Ng et al., 2002).

Functional immaturity of segments of the HPA-axis in preterm infants might arise from underdeveloped structures and organs, as delays in growth and ripening of structures (e.g., lungs, brain; Bolt et al., 2002). An underdeveloped HPA-axis in preterm infants might cause a hampered postnatal hormonal stress-response, and not being able to cope with an extremely stressful environment, such as the neonatal intensive care unit (NICU; Bolt et al., 2002; Finken et al., 2017). The result is a shortage of cortisol right after birth which is referred to as relative adrenal insufficiency (RAI) or adrenal insufficiency of prematurity. RAI is thought to play a role in common neonatal complications of preterm infants, such as hypotension and hypoglycemia (Finken et al., 2017; Ng, 2016). Other studies show a similar reduction in cortisol response in preterm infants (32 weeks gestational age) during their stay in the NICU (Grunau et al., 2005). One recent prospective observational study of preterm infants (<32 weeks gestational age); however, suggests that the adrenal insufficiency resolves in children with RAI as the age of the child increases (Masumoto et al., 2019).

At birth, painful, high-intensity stimuli, absence of maternal care and complications such as infections, hypoglycemia, asphyxia, and hypothermia are thought to negatively impact neurodevelopment and the HPA-axis (Behrman & Butler, 2006; Chau et al., 2017). Neonatal procedural pain-related stress was associated with lower cortisol levels, with effects found primarily in boys (Brummelte et al., 2015). Recently, it has also been found that a stressful/acute delivery, like an emergency cesarean delivery (C-section), is associated with lower infant cortisol levels (Martinez et al., 2020). This may arise from increased cortisol levels in the mother due to prolonged periods of stress, which can influence the HPA-axis stress system in the unborn child through the placenta (Jahnke et al., 2021). Secondly, the clinical indications for doing a C-section, like preeclampsia, are often related to conditions that are accompanied by high levels of anxiety and stress, resulting in prolonged increases of cortisol levels (van Esch et al., 2020).

Malfunction of the HPA-axis due to early-life stress is strongly involved in stress disorders, anxiety, depression, and cognitive disorders (Juruena et al., 2020). There is evidence that children and young adults born prematurely (<32 weeks gestational age) show blunted cortisol responses to (psychological) stress (Finken et al., 2017; Kaseva et al., 2014; Stoye et al., 2022). A longitudinal cohort study showed that neonatal stressful events in prematurely born children (between 24–32 weeks gestational age) were associated with lower cortisol levels and that they show more problems with sensory processing at 4 years old (McLean et al., 2021), suggesting a relationship between cortisol and sensory problems in prematurely born children. However, consistent evidence on cortisol values in children born prematurely is lacking, as intra-individual variation in cortisol-values was high (Zwimpfer et al., 2022).

Whereas evidence on basal cortisol levels is not clearcut, several recent studies show flattened cortisol-responses to (psychological) stress in both infants (4 months) and children (<9 years) born extremely premature or SGA (Brummelte et al., 2015; Hwang et al., 2019; Stoye et al., 2022). Considering the relationship between prematurity, stress and cortisol levels, the aim of this preliminary study is to investigate the influence degree of prematurity (weeks of gestation) and delivery route (C-sections versus vaginal birth) on cortisol levels in children, as a function of their HPA-axis activity. It is hypothesized that lesser weeks of gestation will lead to lower cortisol levels and C-section delivery will likewise add to lower cortisol levels.

## Methods

### Study Population and Selection

This is a cross-sectional study design in which cortisol measurements were done in clinical follow-ups of children with a complicated birth (such as prematurity, dysmaturity or VLBW). Exclusion criteria for participation in this study were systemic administration of corticosteroids, major illness, neurological lesions/deficits, genetic syndromes, and a diagnosis with a major systemic disease or malformations. Test subjects were asked not to use inhalation corticosteroids on the day that the cortisol sample was collected. After applying exclusion criteria, 38 participants were included for this study. Only one child was included in the case of a twin or triplet.

If there was more than one cortisol sample found in patients files, the most recent one was chosen. Electronic patient files were systematically screened for specific variables, like age at time of blood sampling, sex, weeks of gestation, birth weight, season of sample collection, childbirth delivery method, APGAR scores, duration of postnatal hospital stay, if corticosteroids (for lung maturation) were administered during and after pregnancy, health status of parents, peripartum complications, use of medication, and current behavioral, emotional, and neurodevelopmental status of children. All primary caregivers signed a written informed consent for collecting blood during a follow-up session and the study was approved by the ethical committee of the Erasmus University Medical Center, Rotterdam. In total, 38 participants were included in this study.

### Sampling and Cortisol Assay

Participants were recruited at a multidisciplinary pediatric center (Het Kleine Heldenhuis, located in Rotterdam, the Netherlands) connected to the Saint Franciscus hospital and Erasmus University Medical Center hospital in Rotterdam, the Netherlands. This center offers both specialized clinical, pediatric, psychological, social, and pedagogic care for children (and their families) with a complicated start of life, such as preterm (age <37 weeks) or SGA birth. Blood samples were collected between February 1^st^ 2021 and November 1^st^ 2021 for total serum cortisol measurements by the handling pediatrician and after informed consent by the primary caregiver(s) of the child. Blood samples were directly sent to the hospital laboratory of the Saint Franciscus hospital or stored at −20°C and send the following day.

Serum cortisol was determined with a solid-phase radio immune assay RIA from DRG Instruments GmbH, Marburg, Germany. Concentrations were then assessed by an isotope dilution liquid chromatography method and analysis was performed at the laboratory of Saint Franciscus hospital. Inter- and intra-assay coefficients of variation were less than 10% over a concentration range between 30–800 nmol/L.

### Cortisol Analysis

**Figure 1.**
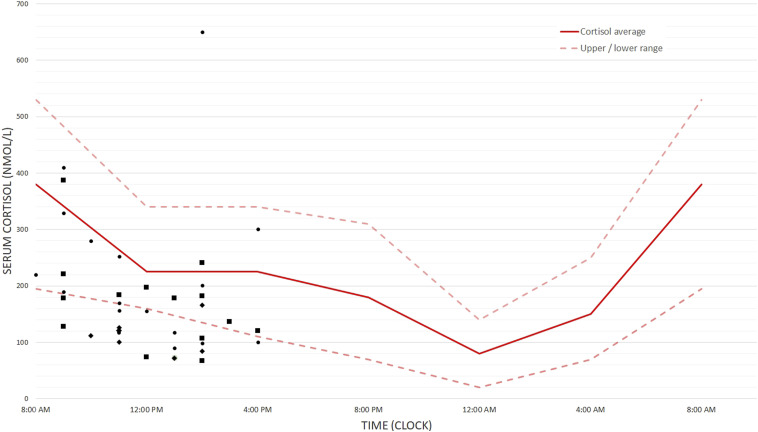
Cortisol measurements in serum blood of participants plotted in the cortisol curve as adapted from Knutsson et al., 1997. Symbols stand for the cortisol measurements in this study, symbols: Moderate to late premature (32–36 weeks), Very premature (28–32 weeks), Extremely premature (<28 weeks).

Our pediatric center specializes in families with a complicated birth, and generally does not receive families with term-born children, so there was no access to a control group. However, standard cortisol curves have been described repeatedly in contemporary scientific literature. Therefore, as controls for the cortisol measurements, blood cortisol reference values were found in 2 different sources of literature (Knutsson et al., 1997; Marshall et al., 2020), which were neither sex- or age specific. The values between the 5^th^ and 95^th^ percentiles for children (age range 2.2–18.5 years old) in the article by Knutsson et al. were used as the reference range, as this study included the age groups of interest. The reference curve for all children was then plotted with lower and upper ranges with the single cortisol measurements of the present study fitted into this curve (see symbols in Figure 1). Cortisol samples were collected at different times, so this had to be controlled for. Secondly, a correction for age was done, so appropriate ages were compared to each other. Cortisol was collected from only one child out of the twins or triplets in this study. Then, the difference between the cortisol measurement for each participant and the average reference value (uninterrupted line) at that specific timepoint was calculated. These values were used for further analyses of cortisol levels and are referred to as ΔCort. A negative ΔCort portrays a cortisol value that is lower than average, whereas a positive ΔCort portrays a value that is higher than average.

### Statistical Analysis

Prior to all analyses, the Kolmogorov-Smirnov test was done to check for assumption of normality distribution of variables. Based on the literature, we included sex, delivery route (C-section vs. vaginal), and degree of prematurity (number of weeks of gestation) to predict cortisol levels in a hierarchical linear regression analysis. For descriptive variables, lambdas were calculated before entering variables into the linear regression. IBM SPSS statistics version 28.0.1.0 was used for statistical analysis.

## Results

Table 1 shows the birth characteristics of the children and their degree of prematurity. Age of the children was between 3 – 9 years, sex ratio was 1:1; 34% were born twins and 3% were born triplets (but cortisol was only taken from one child out of a twin or triplet). A little over half of the children were born through C-section (53%) and the rest were delivered through the vaginal route. Sixteen percent were born extremely preterm (<28 weeks), 37% very preterm, and 47% moderate to late preterm.

**Table 1.** Birth Characteristics of Participants.

Degree of prematurity (in categories)							
		Extremely Preterm (<28 weeks) *n = 6*		Very Preterm (28–32 weeks) *n = 14*		Moderate to late Preterm (>32–36 weeks) *n = 18*	
		*N* (%)	M (SD)	*N* (%)	M (SD)	*N* (%)	M (SD)
Sex	Female	3 (50.0%)		6 (42.9%)		10 (55.6%)	
	Male	3 (50.0%)		8 (57.1%)		8 (44.4%)	
Age (years)			6.33 (2.25)		5.30 (2.31)		6.58 (2.54)
BMI (kg/m^2^)			17.51 (4.97)		14.43 (1.37)		15.65 (2.74)
Gestational age (weeks)			26.50 (1.38)		29.20 (1.32)		34.50 (1.62)
Birth weight (grams)			810.00 (186.68)		1016.00 (287.99)		1901.82 (546.14)
Delivery route	Vaginal	1 (16.7%)		5 (35.7%)		12 (66.7%)	
	C-section	5 (83.3%)		9 (64.3%)		6 (33.3%)	
Duration hospital stay (days)			96 (22)		66 (24)		24 (18)
APGAR at 1 minute			3 (2)		6 (3)		8 (2)
APGAR at 5 minutes			8 (1)		8 (2)		9 (1)
Multiple birth	Single	4 (66.7%)		8 (57.1%)		11 (61.1%)	
	Twin	2 (33.3%)		6 (42.9%)		5 (27.8%)	
	Triplet	0 (.0%)		0 (.0%)		1 (11.1%)	

*Note.* BMI, body mass index; SD, standard deviation, M, mean; *N*, number.

Assumption of normality of the ΔCort variable was checked with the Kolmogorov-Smirnov test, and the null hypothesis of normal distribution was retained (*p* = .20). Associations between dependent and independent variables were checked with crosstabulation. Lambda was .36 for birth delivery route and ΔCort, .24 for sex and ΔCort and .61 for gestational age (in weeks) and ΔCort.

### Degree of Prematurity (Weeks of Gestation) and Delivery Route

Hierarchical linear regression analysis was performed to determine if degree of prematurity (in weeks of gestation), birth delivery route and sex contributed to a difference in cortisol (ΔCort) in children born prematurely. We did a hierarchical regression, in the first step we entered sex, in the second step degree of prematurity (in weeks of gestational age) and in the third step birth delivery route as independent variables for ΔCort. Sex was not associated with ΔCort in the regression analysis (model 1), degree of prematurity was not associated to ΔCort, but it did show a trend (model 2). Finally, delivery route showed a negative association with ΔCort (*ß* = −.42, *p =* .028), with an explained variance of 34% (model 3). This meant that delivery by C-section was associated with lower cortisol levels. All regression statistics are summarized in Table 2.

**Table 2.** Regression Analysis Between Sex, Degree of Prematurity (Weeks of Gestation), Birth Delivery Route and Dependent Variable ΔCort.

	*F*	*B*	*SE B*	*β*	t	*p*	R^2^	R^2^ change
**Model 1**	2.70						.09	.09
Sex		69.28	42.20	.30	1.64	.112		
**Model 2**	3.07						.19	.10
Sex		66.08	40.60	.29	1.63	.116		
Degree of prematurity		9.59	5.34	.32	1.80	.084*		
**Model 3**	4.21						.34	.14
Sex		41.94	38.93	.18	1.08	.291		
Degree of prematurity		5.56	5.23	.22	1.37	.204		
Birth delivery route		−100.9	43.31	−.42	−2.33	.028**		

Regression analysis statistics. *B,* unstandardized regression coefficient; *β,* standardized regression coefficient; SE, standard error.

*p < .10, **p < .05, ***p < .01.

## Discussion

This preliminary study investigated cortisol levels in children born prematurely and important correlates of a premature birth. We hypothesized that degree of prematurity and birth delivery route would be associated to differences in cortisol levels. However, only an association was found between C-sections and cortisol levels as opposed to vaginal birth. Degree of prematurity (weeks of gestation) did show a trend towards lower cortisol levels, but this was not significant. In addition, cortisol levels were generally lower than the average reference curves, suggesting that children born prematurely have lower cortisol levels. Blunted (flat) cortisol-responses in both infants (4 months), children, and young adults born extremely premature might lead to an inadequate regulation of psychological stress, resulting in both internalizing and externalizing behavioral problems, as well as sensory processing disorders later in life (Bagner et al., 2010; Brummelte et al., 2015; Kaseva et al., 2014; McLean et al., 2021; Pyhälä et al., 2017; Stoye et al., 2022).

Unfortunately, we did not have hospital data with clinical indications for why C-sections were performed. There could have been several clinical indications for this, like preeclampsia, the hemolysis, elevated liver enzymes, low platelets (HELLP) syndrome, chronic diabetes, or hypertension (Butwick et al., 2015; Souza et al., 2016). All these conditions may lead to severe maternal stress during the last period of pregnancy, resulting in prolonged increases of cortisol levels (van Esch et al., 2020). Maternal stress can cause permanent damage to the stress-regulation system in children (Fogelman & Canli, 2018; Naber, 2019). The HPA-feedback system is thought to be coregulated by mother and infant in the first phase of life and is heavily influenced by early life stress through epigenetic processes (Provenzi et al., 2019; Sheng et al., 2021). In addition, a particularly stressful event, like an emergency C-section, influences the stress-regulation system, as mothers need more time to recover and might feel traumatized (Tomsis et al., 2021), disabling the mother to down-regulate stress in the infant (Beijers et al., 2014). In addition, mothers are at increased risk for post-partum depression after a C-section delivery, which might lead to prolonged decreased (physical) contact between mother and infant (Xu et al., 2017). The finding in the current study that a C-section delivery leads to lowered cortisol is supported by 2 other recent studies (Martinez et al., 2020; Słabuszewska-Jóżwiak et al., 2020). However, these studies looked at differences observed in infants, shortly to several months after birth and did not study persisting effects of a C-section delivery beyond early infancy and into childhood. Therefore, the finding in this study is very relevant to the knowledge about HPA-functioning in prematurely born children later in life.

A higher degree of prematurity (younger gestational age) showed a trend towards lower serum cortisol. This is supported by other studies that showed blunted cortisol levels in infants, children, and young adults with very low birth weight (VLBW) or who were born prematurely (Brummelte et al., 2015; Kaseva et al., 2014; McLean et al., 2021; Stoye et al., 2022). It has been suggested that this might be caused by prolonged exposure to stress, resulting in a hypoactive HPA-axis (Kajantie & Räikkönen, 2010). From a biological perspective, there is developmental and functional immaturity of units of the HPA axis in preterm infants and subsequent disturbed programming in children born very-extreme prematurely that might lie at the core of a prolonged HPA axis hypoactivity (Bolt et al., 2002; Finken et al., 2017; Ruys et al., 2017).

In addition, infants born at a lower gestational age are usually exposed to more stressors directly postnatally (e.g., painful interventions, maternal separation) for a longer period of time. Greater neonatal procedural pain-related stress was associated with lower cortisol levels, with effects found primarily in boys (Brummelte et al., 2015). In contrast, no difference in sex was found in the current study. This might be due to the small sample size in our study.

Based on the current findings, the programming of the HPA-axis is likely to be interrupted due to neonatal procedural pain-related stress, leading to an altered activity of the HPA-axis later on in childhood (Ruys et al., 2017; Stoye et al., 2022). It is known that prematurity is a risk factor for behavioral, emotional, and developmental problems later on in childhood and adolescence (Frey & Klebanoff, 2016). Also, it affects cortisol and maternal interactive behavior (Brummelte et al., 2011), and this prolonged exposure to stress could be a plausible explanation for a hypoactive HPA-axis. Modifying the HPA-axis at early infancy is essential for the feedback system and stress regulation later in life. This could be improved by high quality mother-child interaction immediately after birth. For instance, Neu et al. (2009) demonstrated how the difference in salivary cortisol levels between mother and infants decreases after a 60-min holding-session (e.g., Kangaroo-care), suggesting a coregulation of the HPA-axis by mother and her preterm infant.

A couple of limitations of this study should be mentioned. Firstly, the use of serum cortisol samples instead of diurnal curves. Cortisol does not only have a diurnal variation, but it is also released in a pulsatile manner (Lee et al., 2015). This makes it difficult to interpret single measurements, especially when not compared to a reference control group. But the combination of the present results with the reference cortisol values can be considered some control for this. Because we did an age-specific correction, cortisol of children were compared to the same ages. However, we do acknowledge that including a control group would have increased robustness of the findings. In addition, some children were using inhalation corticosteroids for lung problems (e.g., asthma), which is a well-known problem in children born prematurely and this may have caused a bias in results. However, recent studies have found no suppression of basal cortisol production in children on inhalation corticosteroids as compared to controls (Smit et al., 2017). The current study clearly invites more research on HPA-axis activity in children born prematurely. Future studies on this topic should include larger case–control studies, more clinical data and also inclusion of diurnal cortisol profiles in children born prematurely.

In conclusion, our preliminary study shows serum cortisol values may be dependent on important correlates of preterm birth. Although results should be interpreted with caution, an association was found between C-section delivery with lower cortisol levels. Clinically underlying factors leading up to the indication for performing a C-section are potential explanations for this. Therefore, more research is needed into these factors. These results might have important clinical implications, because early-life stress and an HPA-axis disturbance could lead to behavioral, emotional, and developmental problems later on in childhood and adolescence.
